# Effects of Temperature and Carbon-Nitrogen (C/N) Ratio on the Performance of Anaerobic Co-Digestion of Dairy Manure, Chicken Manure and Rice Straw: Focusing on Ammonia Inhibition

**DOI:** 10.1371/journal.pone.0097265

**Published:** 2014-05-09

**Authors:** Xiaojiao Wang, Xingang Lu, Fang Li, Gaihe Yang

**Affiliations:** 1 College of Agronomy, Northwest A&F University, Yangling, Shaanxi, People's Republic of China; 2 School of Chemical Engineering, Northwest University, Xian, Shaanxi, People's Republic of China; National Center for Biotechnology Information (NCBI), United States of America

## Abstract

Anaerobic digestion is a promising alternative to disposal organic waste and co-digestion of mixed organic wastes has recently attracted more interest. This study investigated the effects of temperature and carbon-nitrogen (C/N) ratio on the performance of anaerobic co-digestion of dairy manure (DM), chicken manure (CM) and rice straw (RS). We found that increased temperature improved the methane potential, but the rate was reduced from mesophilic (30∼40°C) to thermophilic conditions (50∼60°C), due to the accumulation of ammonium nitrogen and free ammonia and the occurrence of ammonia inhibition. Significant ammonia inhibition was observed with a C/N ratio of 15 at 35°C and at a C/N ratio of 20 at 55°C. The increase of C/N ratios reduced the negative effects of ammonia and maximum methane potentials were achieved with C/N ratios of 25 and 30 at 35°C and 55°C, respectively. When temperature increased, an increase was required in the feed C/N ratio, in order to reduce the risk of ammonia inhibition. Our results revealed an interactive effect between temperature and C/N on digestion performance.

## Introduction

Anaerobic digestion is an effective way of converting agricultural waste into biogas that can be used to generate energy, which is especially efficient in rural western China. In the past decade, this technology has received great attention in both scientific research and practice. However, the efficiency of anaerobic digestion may be limited by inadequate amount and diversity of waste from a single resource, which is insufficient for large-scale digesters, as well as the drawbacks of using single substrates, such as improper carbon-nitrogen (C/N) ratios, low pH of the substrate itself, poor buffering capacity, and high concentrations of ammonia [1], [2], [3]. Therefore, co-digestion of mixture substrates for biogas production has recently attracted more interest.

Co-digestion of various biosolid wastes, a process that utilizes the nutrients and bacterial diversity in those wastes to optimize the digestion process, is an attractive approach for improving the efficiency of biotransformation [4]. A primary advantage of co-digestion is that it could efficiently balance feedstock carbon and nitrogen and a balanced C/N ratio of feedstock is likely to improve methane production. An early study conducted by Wu *et al*. revealed that swine manure co-digested with corn stalks at a C/N ratio of 20 obtained increased cumulative biogas production up to 11-fold and increased cumulative net methane volume up to 16-fold, when compared to swine manure digested alone [5]. Recent study by Wang *et al.* also suggested that co-digestion of dairy manure, chicken manure and wheat straw, had better digestion performance with stable pH and low concentrations of total ammonium nitrogen (TAN) and free ammonia (FA) at adjusted C/N ratios of 25 and 30 [6]. Similar observations were also reported by Hills for dairy manure, demonstrating that the greatest methane production was achieved when the C/N ratio was adjusted to 25 using glucose [7]. By optimizing the substrate C/N ratio, co-digestion of wastes of different C/N characteristics can greatly enhance the efficiency of biogas digestion.

Although many studies indicated that the optimal C/N ratios in methane fermentation were 25∼30 [8], [9], [10], the depletion of carbon and nitrogen could be affected by operating conditions, such as temperature, resulting in the occurrence of inhibitory effects. It has been reported that the high FA concentration could inhibit thermophilic more seriously than mesophilic digestion [11], [12], [13]. A decrease in operating temperature from 60°C to 37°C in anaerobic digesters with a high ammonia concentration provided relief from FA inhibition, leading to increase in biogas yield [14], [15]. FA concentration under mesophilic digestion is already inhibitory in the range of 80∼150 mg L^−1^ at a pH of 7.5 [16], [17], [18]. However, under thermophilic conditions, when the concentration of FA was increased to 620 mg L^−1^ in the ammonia toxicity test, a gradual decrease of 21% was observed in biogas [19]. Another study also indicated that thermophilic flora tolerated at least twice as much FA compared to mesophilic flora [20]. Because the concentrations of TAN and FA originally depend on the content of organic nitrogen in the reactor and on C/N ratios, the indicator of substrate carbon and nitrogen content may also interact with temperature and that interaction results in different concentrations of ammonia and FA, as well as inhibitory effects.

Base on previous studies mentioned above, there are interactive effects between temperature and ammonia in the digestion process and the digestion efficiency is dramatically affected by the temperature and C/N ratio. Thus, to investigate this interaction, we first examined the effect of a series of temperatures on the mixtures of certain ratios of C/N (25), and secondly, compared the digestion performance of mixtures with a series of C/N ratios by adjusting the proportions of each substrate, dairy manure (DM), chicken manure (CM) and rice straw (RS) under mesophilic and thermophilic conditions.

## Materials and Methods

### Substrate characteristics

DM and CM were collected from a livestock farm located in Yangling, China. RS was obtained from a local villager. Before being put into the reactor, the air-dried RS was cut into pieces (2∼3 cm). The substrates were individually homogenized and subsequently stored at 4°C for further use. The chemical characterization of each substrate tested in this study is shown in Table 1. All samples were collected and tested in triplicate, and the averages of the three measurements are presented.

**Table 1:** Chemical characteristics of raw materials used in this study.

Substrate	a TS content/%	VS content/%	pH	Total carbon/g kg^−1^VS	Total Kjeldahl nitrogen/g kg^−1^VS	C/N
DM	15.8±0.34	81.5±1.41	7.26±0.03	65.8±1.19	2.96±0.05	22.2±0.22
CM	29.9±0.67	65.3±1.26	6.93±0.11	58.6±1.77	6.11±0.08	9.6±0.16
RS	89.2±1.59	92.3±1.34	-	328±5.67	6.34±0.11	51.7±1.62

a ±shows the standard error.

### Ethics statement

The collections of DM and CM were permitted by livestock farms belonging to ‘Besun’ group in Yangling, China. The RS was provided voluntarily by a local villager in Qishan, Baoji, China. The inoculum was obtained from a household biogas digester in a biogas demonstration village named Cuixigou in Yangling and the collection was permitted by the hosts. The all experimental procedures conformed to the regulations established by the Ethics Committee of the Research Center of Recycle Agricultural Engineering and Technology of Shaanxi Province, China.

### Experimental design and set-up

Experiment 1: Three mixture sets were investigated in this experiment: set A (DM+ RS), set B (CM+RS), and set C (DM+CM+RS). For set A and set B, the C/N ratio was 25, achieved by adjusting the DM/RS or CM/RS ratio. For set C, based on a DM/CM ratio of 1∶1, multi-component substrates were prepared by adding RS to the DM-CM mixtures in order to adjust the C/N ratio to 25. The proportions of all substrates in each mixture were in a volatile solid (VS) state. The operation temperatures were 20, 30, 40 (mesophilic), 50, and 60°C (thermophilic), respectively.

Experiment 2: For all mixture sets, RS was added into the DM-CM mixtures with a VS ratio of 1∶1, in order to adjust the C/N ratio to selected levels. C/N ratios of 15, 20, 25, 30 and 35 were selected in tests at a temperature of 35°C, but ratios of 20, 25, 30, 35 and 40 were selected in tests at a temperature of 55°C.

The initial VS ratio of substrate to inoculum was kept at 1∶2 for all experimental setups. Each reactor had a 1 L capacity and contained 600 mL of total liquid, including 200 mL of inoculum and mixed substrate of 15gVS/L. The inoculum used for digestion at 20, 30, 35 and 40°C was digested cattle manure, taken from a lab-scale reactor operated at 35°C with a hydraulic retention time (HRT) of 15 days. Additionally, digestion at 50, 55 and 60°C was inoculated with digested cattle manure from the lab-scale reactor operated at 55°C with a HRT of 15 days. A control with only inoculum was used to determine biogas production due to endogenous respiration. Each treatment was performed in triplicate. All reactors were tightly closed with rubber septa and screw caps. The headspace of each reactor was flushed with nitrogen gas for about 3 min to assure anaerobic conditions prior to starting the digestion tests. To provide mixing of the reactor contents, all reactors were shaken manually for about 1 min, once a day prior to measurement of biogas volume.

### Analytical techniques

Total solids, VS, pH, total Kjeldahl nitrogen (TKN), and total ammonium nitrogen (TAN) analysis were performed according to APHA Standard Methods [21]. Total organic carbon was determined by the method described by Cuetos *et al.* [22]. For all treatments, FA concentration was calculated in accordance with Hansen *et al*. [23]. The volume of biogas was measured by displacement of water. Methane content in the produced biogas was analyzed with a fast methane analyzer (Model DLGA-1000, Infrared Analyzer, Dafang, Beijing, China). The C/N ratio was determined by dividing the total organic carbon content by the total nitrogen content, according to the following equation. 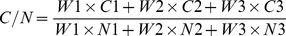



Where W1, W2 and W3 were the VS weight in a single substrate in the mixture, C1, C2 and C3 were the organic carbon content (g kg^−1^VS) in each substrate and N1, N2 and N3 were the nitrogen content (g kg^−1^VS) in each substrate.

## Results

### Effects of temperature on the performance of anaerobic co-digestion based on experiment 1

Increased temperature resulted in pH increases in all three mixtures (Table 2). The pH values in digesters at 20°C, with average values of 6.12, 5.42 and 5.92 in the mixtures of DM+RS, CM+RS and DM+CM+RS, respectively, were far lower than those under other temperatures. From 30 to 60°C, the average pH values were in the range of 6.42 ∼7.82.

**Table 2:** Effects of temperature on pH value in anaerobic co-digestion with a C/N ratio of 25.

Temperature (°C)	DM+RS		CM+RS		DM+CM+RS	
	a Average	Final	Average	Final	Average	Final
20	6.12±0.13	6.64±0.14	5.42±0.11	5.01±0.07	5.92±0.10	6.58±0.11
30	6.89±0.15	7.12±0.16	6.42±0.03	6.92±0.02	7.11±0.11	7.35±0.14
40	7.21±0.09	7.44±0.13	7.19±0.08	7.38±0.12	7.48±0.13	7.67±0.11
50	7.58±0.11	7.61±0.13	7.66±0.12	7.79±0.07	7.56±0.12	7.74±0.03
60	7.69±0.15	7.88±0.15	7.82±0.10	8.11±0.11	7.72±0.16	7.92±0.13
bLSD_0.05_ = 0.47 cLSD_0.05_ = 0.61						

a ±shows the standard error

b LSD value at the 5% level based on all average values from three mixture sets at all operation temperatures

c LSD value at the 5% level based on all final values from three mixture sets at all operation temperatures

A linear correlation between TAN and temperature (20 – 60°C) was observed and the highest TAN value was 1,261 mg L^−1^ in the mixture of CM+RS at 50°C (Table 3). The relationship between FA (Y, mg L^−1^) and temperature (T, °C) was evaluated by the following equations: Y = 0.0302e^1.82T^ in the mixture of DM+RS, Y = 0.0216e^2.0T^ in the mixture of CM+RS and Y = 0.101e^1.65T^ in the mixture of DM+CM+RS. On average, the mixture of DM+CM+RS had significantly higher TAN and FA concentrations than the mixture of DM+RS, but was lower than the mixture of CM+RS (Tables 3 and 4).

**Table 3:** Effects of temperature on total ammonia content in anaerobic co-digestion with a C/N ratio of 25.

Temperature (°C)	DM+RS		CM+RS		DM+CM+RS	
	a Average	Final	Average	Final	Average	Final
20	182.3±3.9	229.3±4.8	495.2±7.2	532±9.2	477±8.3	521±1.88
30	260.2±5.8	518.5±8.5	552.5±2.0	674±14.3	531±10.2	778±12.8
40	421.2±7.5	772.7±13.4	768.4±12.6	995±22.2	737±10.7	921±17.7
50	541.5±9.4	968.6±18.7	938.3±14.8	1261±18.3	869±3.1	1116±16.3
60	593.6±11.4	1052.4±18.8	951.8±12.5	1201±4.8	906±14.9	1256±20.6
bLSD_0.05_ = 52.6 cLSD_0.05_ = 81.9						

a ±shows the standard error

b LSD value at the 5% level based on all average values from three mixture sets at all operation temperatures

c LSD value at the 5% level based on all final values from three mixture sets at all operation temperatures.

**Table 4:** Effects of temperature on free ammonia content in in anaerobic co-digestion with a C/N ratio of 25.

Temperature (°C)	DM+RS		CM+RS		DM+CM+RS	
	aAverage	Final	Average	Final	Average	Final
20	0.1±0.004	0.4±0.009	0.1±0.002	0.9±0.01	0.2±0.001	0.8±0.01
30	1.6±0.04	5.4±0.08	1.2±0.03	4.4±0.06	5.4±0.09	13.8±0.1
40	12.7±0.5	31.4±0.6	17.9±0.8	35.5±1.6	32.8±1.1	63.7±2.1
50	65.4±2.2	101.2±4.8	108.6±3.7	189.2±4.3	82.0±1.8	153.3±3.2
60	142.5±3.6	324.5±4.9	240.7±5.2	479.2±3.2	192.6±3.2	376.7±8.2
bLSD_0.05_ = 23.2 cLSD_0.05_ = 45.6						

a ±shows the standard error

b LSD value at the 5% level based on all average values from three mixture sets at all operation temperatures

c LSD value at the 5% level based on all final values from three mixture sets at all operation temperatures

**Figure 1:**
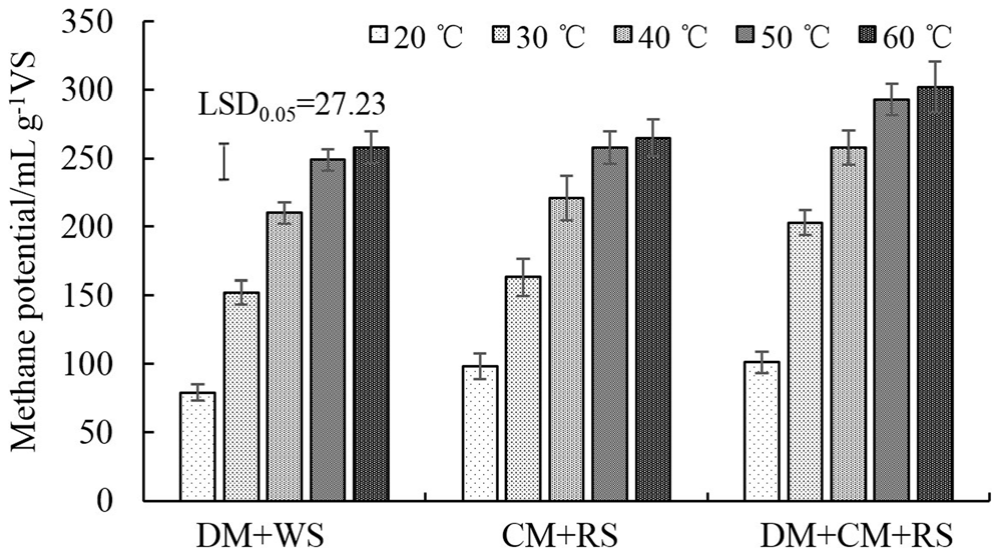
Effects of temperature on methane potential in mixtures with a C/N ratio of 25. Values are presented as the mean ±standard error of three replicates (n = 3). Vertical bars represent LSD at the 5% level.

With the increase of temperature, methane potential continuously increased, but the increasing rate was lower under thermophilic than under mesophilic conditions (Fig. 1). The mixture of DM+CM+RS had a little higher methane potential than the mixtures of DM+RS and CM+RS.

### Effects of C/N ratio on the performance of anaerobic co-digestion based on experiment 2

**Figure 2:**
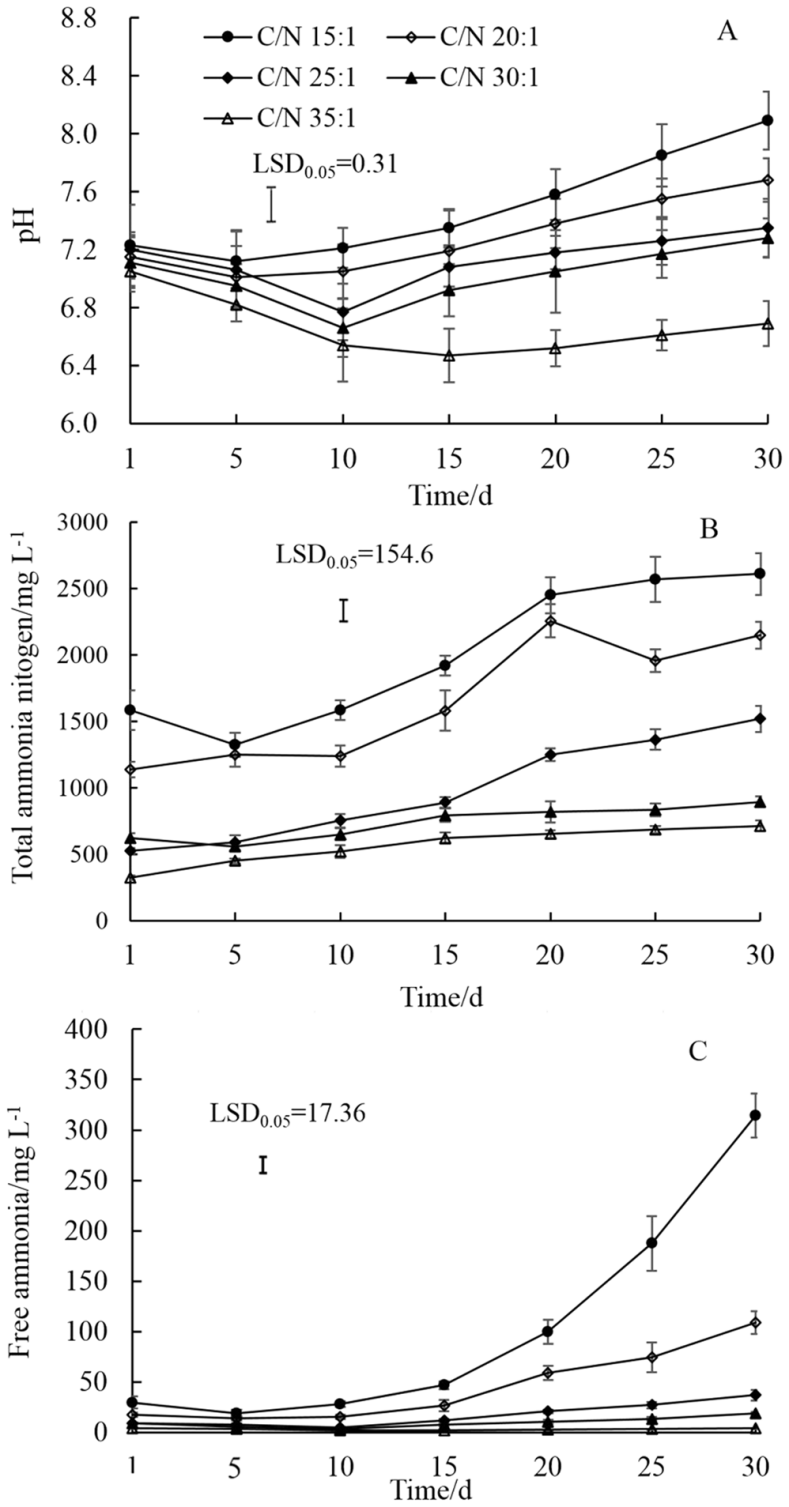
Changes of pH, total ammonium nitrogen, and free ammonia with different C/N ratios in the mixture of dairy manure (DM), chicken manure (CM), and rice straw (RS) in anaerobic co-digestion at 35°C. Values are presented as the mean ±standard error of three replicates (n = 3). Vertical bars represent LSD at the 5% level.

The pH value and the concentrations of TAN and FA were significantly influenced by C/N ratios at 35°C. For digesters with C/N ratios of 15 and 20, the pH values were higher than 7.0 during the whole digestion process, and the final pH values reached to 8.09 and 7.68, respectively (Fig. 2A).The average pH value was as low as 6.67 when the C/N ratio increased to 35. C/N ratios of 25 and 30 resulted in average pH values of 7.12 and 7.02, respectively. In addition, the contents of TAN and FA decreased with increased C/N ratios (Fig. 2B and C). Low C/N ratios of 15 and 20 resulted in TAN and FA concentrations as high as 2610, 2258 mg L^−1^ and 314, 108 mg L^−1^, respectively. Treatments with C/N ratios of 25, 30 and 35 resulted in low and stable TAN and FA during the anaerobic process. The average concentrations of TAN were 985, 739 and 568 mg L^−1^ when C/N ratios were of 25, 30, and 35, respectively and the average concentrations of FA were 9.1, 7.5 and 2.2 mg L^−1^ when C/N ratios were of 25, 30, and 35, respectively.

**Figure 3:**
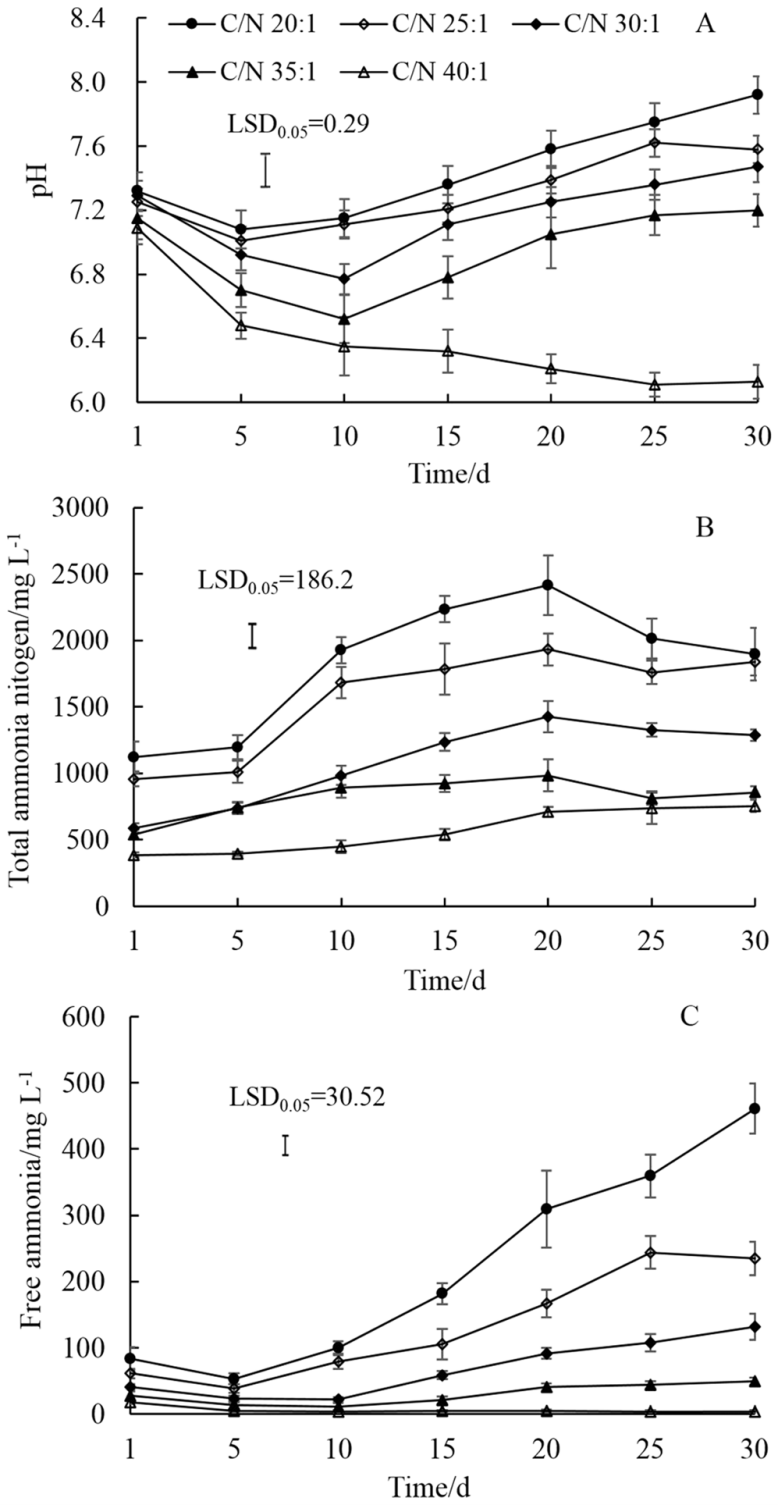
Changes of pH, total ammonium nitrogen, and free ammonia with different C/N ratios in the mixture of dairy manure (DM), chicken manure (CM), and rice straw (RS) IN anaerobic co-digestion at 55°C. Values are presented as the mean ±standard error of three replicates (n = 3). Values are presented as the mean ±standard error of three replicates (n = 3). Vertical bars represent LSD at the 5% level.

Under 55°C, pH values were between 7.0 and 7.92 in treatments with C/N ratios of 20 and 25. Stable pH values around 7.0 were observed when C/N ratios were of 30 and 35. When the C/N ratio was increased to 35, the pH value was lower, at around 6.2 (Fig. 3A). The concentrations of TAN in treatments with C/N ratios of 20 and 25 increased up to 1500 mg L^−1^ by day 10 and reached peaks as high as 2415 and 1932 mg L^−1^, respectively (Fig. 3B). FA increased continuously in digestion with final concentrations of 461 and 235 mg L^−1^ when C/N ratios were of 20 and 25. For C/N ratios between 30 and 40, TAN and FA concentrations were in the range of 430∼1426 mg L^−1^ and 2∼131 mg L^−1^, respectively (Fig. 3B and C).

**Figure 4:**
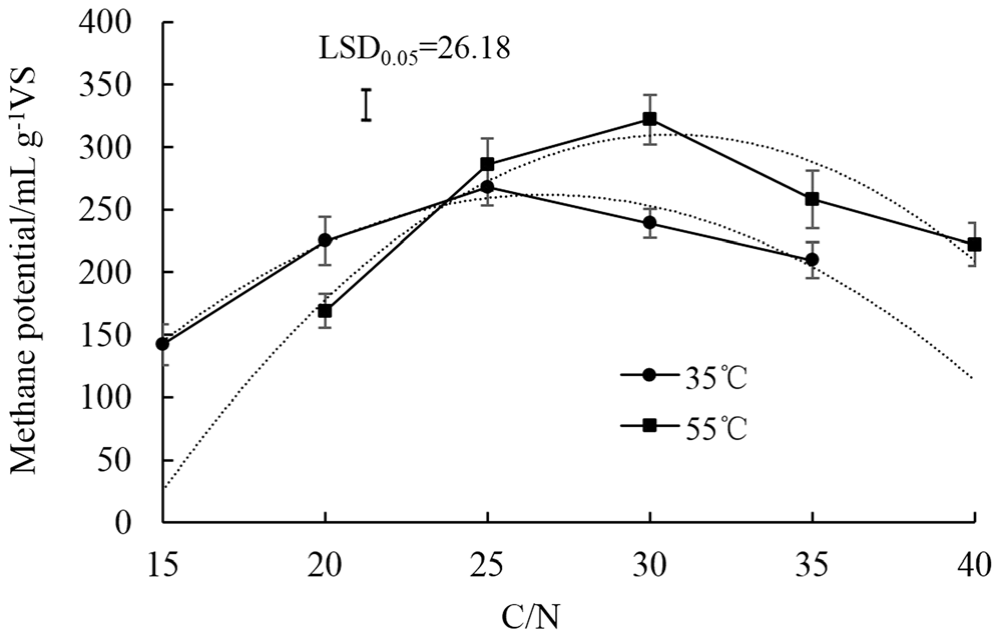
Changes of methane potential with different C/N ratios in the mixture of dairy manure (DM), chicken manure (CM), and rice straw (RS) in anaerobic co-digestion at 35°C and 55°C. The dotted lines were fitting curves for both temperatures. Values are presented as the mean ±standard error of three replicates (n = 3). Vertical bars represent LSD at the 5% level.



Methane potential increased first and then decreased with increases of C/N ratios. The highest methane potential was observed with a C/N ratio of 25 at 35°C with 272 mL g^−1^VS and with a C/N ratio of 30 at 50°C with 286 mL g^−1^VS, respectively (Fig. 4). The quadratic models for methane potential in terms of the C/N ratio as a variable were significant and the equations at 35°C (1) and 55°C (2) were expressed as follows:

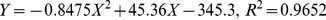
(1)

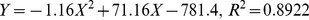
(2)


Where Y was methane potential and X was the C/N ratio. The optimum conditions for maximum methane potential were calculated as a C/N of 26.76 at 35°C and a C/N ratio of 30.67 at 55°C, respectively. Accordingly, the highest methane potential was estimated as 265.7 and 309.9 mL g^−1^ VS.

## Discussion

According to the study by Calli *et al*., ammonia inhibition occurs in the range of 1500∼3000 mg L^−1^ TAN when the pH value is over 7.4 [24]. Then, TAN concentrations of three mixtures were in a safe range below 1261 mg L^−1^ at temperatures between 20 and 60°C (Table 3). Compared with ammonium nitrogen, FA has been suggested as the active component causing ammonia inhibition, since it is freely membrane-permeable [25]. It has been reported that a range between 80 and 150 mg L^−1^ FA was inhibitory for methanogens [16], [26]. In our study, FA concentrations were in this range at 50°C and far higher than 150 mg L^−1^ at 60°C (Table 4), indicating the occurrence of ammonia inhibition. Based on experiment 1, temperature obviously played a greater role in methane production in the range of 20 ∼40°C than in the range of 40 ∼60°C. Methane potentials in three mixtures were an average of 2.49 times higher at 40 than 20°C, but only 1.20 times higher at 60 than at 40°C. And no significant difference was found in methane potential between 50 and 60°C. These results also suggest the existence of an inhibitory effect by ammonia under thermophilic conditions. However, in the anaerobic digestion of organic wastes, it has been reported that methane production was inhibited up to 50% by 220 mg L^−1^ FA at 37°C and by 690 mg L^−1^ FA at 55°C [20]. That is, thermophilic flora tolerated at least twice as much FA as compared to mesophilic flora. The higher methane potential under thermophilic conditions suggested that increased ammonia did not completely inhibit the digestion process and did not offset the advantage of increased temperature in thermodynamics and kinetics, which might result from proper C/N ratios of mixture substrates.

Due to the potential role of the C/N ratio in regulating the inhibitory effects of ammonia, digestions with different C/N ratios were tested in experiment 2 under mesophilic and thermophilic conditions to further obtain optimal C/N ratios with less ammonia inhibition. We found that the mixture of DM+CM+RS had better digestion performance in methane potential than the mixtures of DM+RS and CM+RS (Fig. 1), which might be due to the increased buffering capacity and the synergistic effect, which was inconsistent with the result reported by Wang *et al*. [6]. The mixture of DM+CM+RS was then selected for follow-up studies.

Substrates that have low C/N ratios contain relatively high concentrations of ammonia, exceeding concentrations necessary for microbial growth, and probably inhibiting anaerobic digestion [3], [23]. TAN concentrations were as high as 2500 mg L^−1^ and FA increased up to final concentrations of 314 and 461 mg L^−1^, when the C/N ratio was of 15 at 35°C and was 20 at 55°C, respectively (Figs. 2 and 3). Therefore, methane potential was reduced down to 142 mLg^−1^VS at 35°C and 169 mLg^−1^VS at 55°C, accounting for just 53.0% and 52.5%, compared with their maximum values (Fig. 4). Under both temperatures, with the increase of C/N ratios, TAN and FA concentrations decreased. For example, the average FA concentrations at 35°C were reduced 56.5, 83.7, 90.7 and 97.1% from a C/N ratio of 15 to 20, 25, 30 and 35, respectively. Previous reports suggested that using a feedstock C/N ratio from 27 to 32 promotes steady digester operation at optimum ammonia nitrogen levels and feedstock with a C/N ratio of 32 producing a lower concentration of ammonia nitrogen and FA [25], [27]. Thus, the digestion system was sensitive to the feed C/N ratio and a higher C/N ratio reduced the protein solubilization rate and hence produced lower TAN and FA concentration within the system, which was found to be advantageous.

Ammonia inhibition under mesophilic and thermophilic conditions has been compared in previous studies. It has been observed that an increase in temperature resulted in a reduction of the biogas yield, due to the increased inhibition of FA under higher temperature [14], [15], [28]. In our study, ammonia inhibition occurred with a C/N ratio of 20 at 55°C, whereas a C/N ratio of 15 experienced inhibition at 35°C, suggesting that higher temperature improved the degradation efficiency of organic nitrogen to ammonia nitrogen. However, when C/N ratios were higher than 25, methane potential at 55°C was higher than at 35°C (Fig. 4), indicating higher C/N ratios reduced the risk of ammonia inhibition under thermophilic conditions. Moreover, the optimal C/N ratios were obtained at 26.76 and 30.67 under mesophilic and thermophilic conditions, respectively, by optimizing the quadratic models between methane potential and C/N ratio. These results showed that ammonia inhibition occurring under thermophilic conditions might be avoided by optimizing the C/N ratio in co-digestion of different substrates. However, a very high C/N ratio promotes the growth of methanogen populations that are able to meet their protein requirements and will, therefore, no longer react with the remaining carbon content of the substrate, resulting in a low production of gas.

## Conclusions

This study demonstrated an interactive effect between C/N ratio and temperature on the performance of anaerobic co-digestion of dairy manure, chicken manure and rice straw. Our results suggest that increased temperature from mesophilic to thermophilic conditions resulted in ammonia inhibition, however, this kind of inhibition could be reduced or avoided by increasing the C/N ratio of mixed feedstock to an appropriate level. In anaerobic co-digestion of DM, CM and RS, the optimal C/N level was 26.76 at 35°C and 30.67 at 55°C. Adjusting the proportions of mixture substrates in anaerobic co-digestion to obtain suitable feed characteristics, such as the C/N ratio, pH and nutrients, is an effective way to achieve desired digestion performance.
